# Transcriptome Profiling Associated with *CARD11* Overexpression in Colorectal Cancer Implicates a Potential Role for Tumor Immune Microenvironment and Cancer Pathways Modulation via NF-κB

**DOI:** 10.3390/ijms251910367

**Published:** 2024-09-26

**Authors:** Faisal Alhosani, Burcu Yener Ilce, Reem Sami Alhamidi, Poorna Manasa Bhamidimarri, Alaa Mohamed Hamad, Noura Alkhayyal, Axel Künstner, Cyrus Khandanpour, Hauke Busch, Basel Al-Ramadi, Kadria Sayed, Ali AlFazari, Riyad Bendardaf, Rifat Hamoudi

**Affiliations:** 1 Research Institute of Medical and Health Sciences, University of Sharjah, Sharjah P.O. Box 27272, United Arab Emirates; u20105895@sharjah.ac.ae (F.A.); bilce@sharjah.ac.ae (B.Y.I.); ralhamidi@sharjah.ac.ae (R.S.A.); poorna.manasa@gmail.com (P.M.B.); alaahamad607@gmail.com (A.M.H.); 2 Department of Clinical Sciences, College of Medicine, University of Sharjah, Sharjah P.O. Box 27272, United Arab Emirates; 3 Medical Systems Biology Group, Lübeck Institute of Experimental Dermatology, University of Lübeck, Ratzeburger Allee 160, 23538 Lübeck, Germany; axel.kuenstner@uni-luebeck.de (A.K.); hauke.busch@uni-luebeck.de (H.B.); 4 Forensic Laboratory Department, Sharjah Police Headquarters, Sharjah P.O. Box 1965, United Arab Emirates; 5 Oncology Unit, University Hospital Sharjah, Sharjah P.O. Box 72772, United Arab Emirates; noura.alkhayyal@uhs.ae (N.A.); riyad.bendardf@uhs.ae (R.B.); 6 Department of Hematology and Oncology, University Cancer Center Schleswig-Holstein, University Hospital Schleswig-Holstein, University of Lübeck, 23562 Lübeck, Germany; cyrus.khandanpour@uksh.de; 7 Department of Medical Microbiology and Immunology, College of Medicine and Health Sciences, United Arab Emirates University, Al Ain P.O. Box 15551, United Arab Emirates; ramadi.b@uaeu.ac.ae; 8 Zayed Center for Health Sciences, United Arab Emirates University, Al Ain P.O. Box 15551, United Arab Emirates; 9 ASPIRE Precision Medicine Research Institute Abu Dhabi, United Arab Emirates University, Al Ain P.O. Box 15551, United Arab Emirates; 10 Department of Pathology and Laboratory Medicine, American Hospital Dubai, Dubai P.O. Box 3050, United Arab Emirates; ksayed@ahdubai.com; 11 Mediclinic Welcare Hospital, Dubai P.O. Box 31500, United Arab Emirates; ali.alfzari@mediclinic.ae; 12 Center of Excellence for Precision Medicine, Research Institute of Medical and Health Sciences, University of Sharjah, Sharjah P.O. Box 27272, United Arab Emirates; 13 BIMAI-Lab, Biomedically Informed Artificial Intelligence Laboratory, University of Sharjah, Sharjah P.O. Box 27272, United Arab Emirates; 14 ASPIRE Precision Medicine Research Institute Abu Dhabi, University of Sharjah, Sharjah P.O. Box 27272, United Arab Emirates; 15 Division of Surgery and Interventional Science, University College London, London WC1E 6BT, UK

**Keywords:** *CARD11*, colorectal cancer, tumor immune microenvironment, GSEA, NF-κB pathway

## Abstract

The immune system plays a critical role in inflammation by initiating responses to infections or tissue damage. The nuclear factor-κB (NF-κB) pathway plays a key role in inflammation and innate immunity, as well as other cellular activities. Dysregulation of this well-choreographed pathway has been implicated in various diseases, including cancer. CARD11 is a key molecule in the BCL10-MALT1 complex, which is involved in transducing the signal downstream of the NF-κB pathway. This study aims to elucidate how *CARD11* overexpression exacerbates the prognosis of colorectal cancer (CRC). To identify the cellular pathways influenced by *CARD11*, transcriptomic analysis in both CRC cell lines and patients was carried out on *CARD11*– overexpressed HCT-116 and HT-29 CRC cell lines alongside empty vector-transfected cell lines. Furthermore, a comparison of transcriptomic data from adenoma and carcinoma CRC patients with low- (*CARD11*–) and high-(*CARD11*+) *CARD11* expression was carried out. Whole transcriptomics and bioinformatics analysis results indicate that *CARD11* appears to play a key role in CRC progression. Absolute GSEA (absGSEA) on HCT-116 transcriptomics data revealed that *CARD11* overexpression promotes cell growth and tissue remodeling and enhances immune response. Key genes co-expressed with *CARD11*, such as *EP300*, *KDM5A*, *HIF1A*, *NFKBIZ*, and *DUSP1*, were identified as mediators of these processes. In the HT-29 cell line, *CARD11* overexpression activated pathways involved in chemotaxis and extracellular matrix (ECM) organization, marked by *IL1RN*, *MDK*, *SPP1*, and chemokines like *CXCL1*, *CXCL3,* and *CCL22*, which were shown to contribute to the more invasive stage of CRC. In patient samples, adenoma patients exhibited increased expression of genes associated with the tumor immune microenvironment, such as IL6ST, collagen family members, and CRC transition markers, such as *GLI3* and *PIEZO2,* in *CARD11*+ adenoma patients. Carcinoma patients showed a dramatic increase in the expression of *MAPK8IP2* in *CARD11*+ carcinoma patients alongside other cancer-related genes, including *EMB*, *EPHB6*, and *CPEB4*.

## 1. Introduction

CRC is considered to be the third most common form of cancer, and it has become more prevalent with the advancing age of the population [1]. Recently, there has been a noticeable concern about the changing trend in which the diagnosis of CRC in younger adults is rising annually by around 3% in people under the age of 50. In addition, they all share four common signs of early-onset CRC such as diarrhea, rectal bleeding, abdominal pain, and iron deficiency [2,3].

At the cellular level, cell cycle dysregulation and immune cell infiltration of the colonic epithelium could contribute to CRC in terms of sustained inflammation and an increase in the chance of developing carcinoma [4,5].

The genetic instability and the mutation burden in tumor cells necessitate the development of therapeutic strategies that address the heterogeneity and dynamics within tumors. This is where personalized treatments, such as targeted therapy, come into play for treating tumors by identifying new biomarkers that enable prevention, diagnosis, prognosis, and therapeutics.

NF-κB (nuclear factor kappa-light-chain-enhancer of activated B cells) has a central role in mediating the central organization of inflammatory responses, immune responses, cell survival, and apoptosis. This is achieved by regulating numerous transcription factors, inflammatory cytokines, and molecules related to the intestines. Upon response to various triggers such as pathogens and abnormal cell growth, the NF-κB signaling initiates and orchestrates inflammation processes [6]. Since it has been documented that the NF-κB signaling pathway is constitutively activated in various tumor tissues, few studies have focused on the NF-κB pathway to target cancer as a therapy [7,8]. One of the features of CRC is the dysregulation of the NF-κB pathway, which engages in colonic inflammation [9]. Studies have shown that 66% of CRC cell lines and 40% of human CRC tissues exhibited constitutive activation of NF-κB [10]. Many stimuli activate NF-κB by phosphorylating and ubiquitinating, thus degrading the inhibitory molecules that keep NF-κB subunits in the cytoplasm. The IκB-kinase (IKK) complex is responsible for signal-induced phosphorylation of the inhibitor of κBs (IκBs) [11]. The IKK complex is activated by various stimuli that require the formation of the CARD11-BCL10-MALT1 (CBM) complex in the cytoplasm triggered by stimulation through the T- and B-cell receptors (TCR/BCR) [12].

One of the genes involved in transducing NF-κB signaling via B- and T-cell receptors is the Caspase Recruitment Domain Family Member 11 (*CARD11*) gene. CARD11 is a multi-domain scaffold protein carrying a characteristic caspase-associated recruitment domain [13]. CARD11 is a crucial signal transducer between antigen recognition and the activation of downstream NF-κB in lymphocytes and is an essential signaling molecule in adaptive immune response [14].

**Figure 1:**
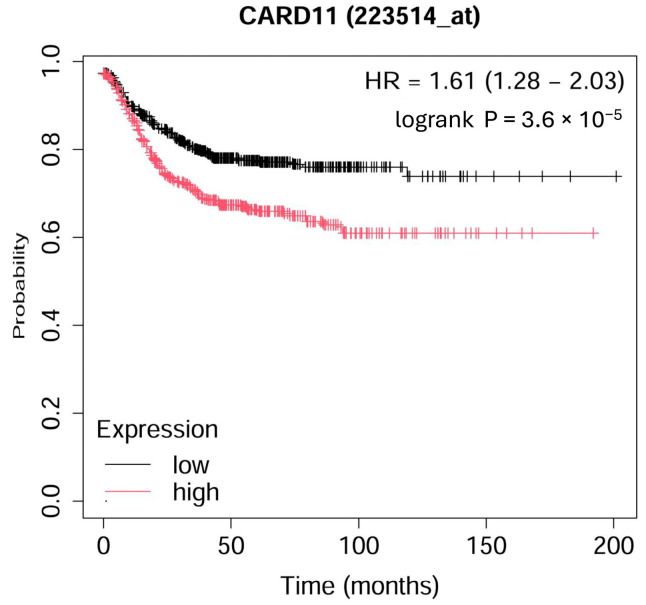
Kaplan–Meier overall survival plot for colorectal cancer patients based on *CARD11* expression. The analysis ran on 1167 patients; 598 patients had high expression, and 569 patients had low expression of *CARD11* (https://kmplot.com (accessed on 17 September 2024)).

The Kaplan–Meier survival curve from microarray-based gene expression data revealed a significant decrease (*p* < 0.001) in survival for colorectal patients with higher expression of *CARD11* (Figure 1).

To the best of our knowledge, this is the first study that attempts to investigate the role of *CARD11* overexpression on the modulation of NF-κB activation in colorectal cancer. Currently, it is unclear whether *CARD11* overexpression exerts an effect on the transcriptomic profiles in colorectal cancer cells. To gain a better understanding of the role of *CARD11* in CRC pathogenesis and, more specifically, how overexpression of *CARD11* can affect the downstream signaling pathways in the pathogenesis of CRC, the present study aims to characterize whole transcriptomic changes associated with the effect of *CARD11* overexpression in CRC cell lines as well as the effect of *CARD11* overexpression in colorectal adenoma and carcinoma patient samples. Understanding the role of *CARD11* in CRC may help in identifying novel diagnostic and therapeutic targets, as well as shed light on some of the potentially novel mechanisms involved in CRC progression via NF-κB dysregulation.

## 2. Results

### 2.1. Overexpression of CARD11 in the HCT-116 and HT-29 CRC Cell Lines Shows a Correlation between mRNA and Protein Levels

**Figure 2:**
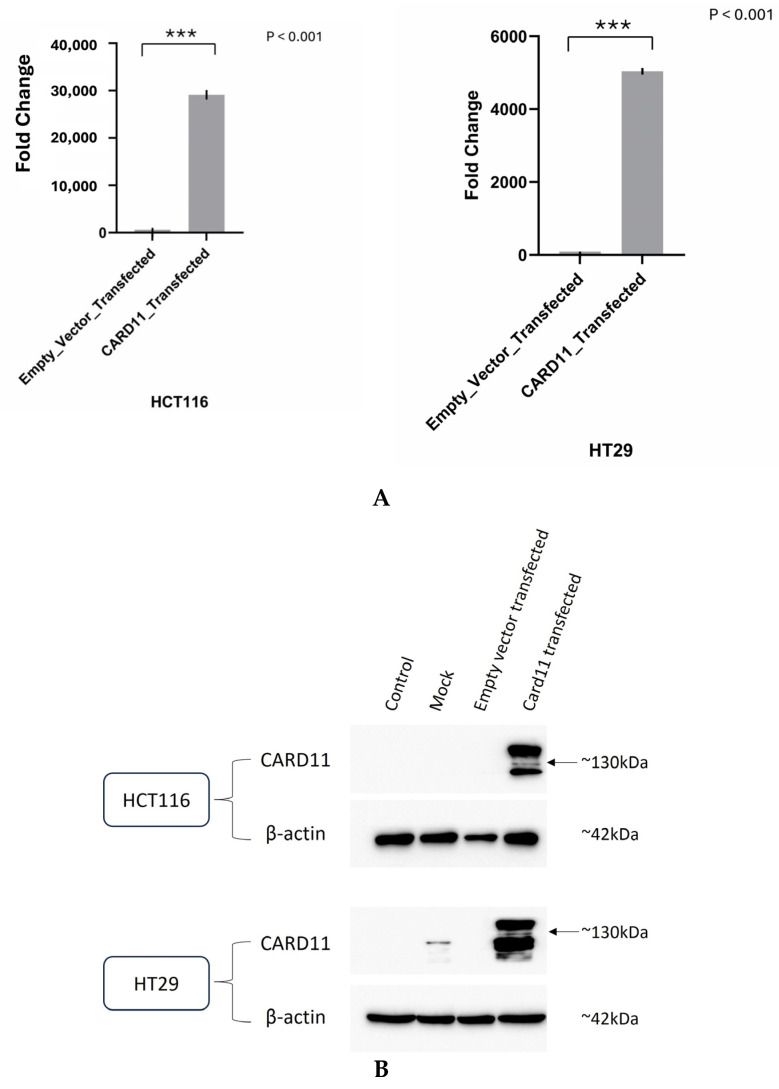
Validation of successful overexpression of *CARD11* in the CRC (HCT-116 and HT-29) cell lines. (**A**) *CARD11* mRNA expression in empty pcDNA3 vector or pcDNA3-*CARD11* transfected HCT-116 and HT-29 cells, as determined by qRT-PCR. Data were normalized to the expression of the housekeeping gene and the 18S rRNA gene, and fold expressions were plotted relative to expression in the empty vector-transfected (control). These data represent the mean ± SD of three independent experiments. *** *p* < 0.001. (**B**) Relative *CARD11* protein expression was determined with a Western blot. Blots were probed with anti-β-Actin antibody as control, confirming equal loading across the lanes.

We aimed to determine if ectopic overexpression of *CARD11* in CRC cell lines induces genome-wide transcriptional changes. Tumors that are well differentiated tend to be less aggressive and grow more slowly. Undifferentiated or poorly differentiated cancer cells look and behave very differently from normal cells in the tissue and tend to be more aggressive [15]. As part of this study, we used two colorectal carcinoma cell lines to identify the cellular pathways modulated by the dysregulation of *CARD11*. HCT116 is an aggressive cell line that does not differentiate, whereas HT29 shows an intermediate ability to differentiate, making it less aggressive than HCT116 [16]. HCT-116 and HT-29 were transiently transfected with either an empty pcDNA3 vector or *CARD11* expression construct. For the HT-29 cell line, 48 h was shown to be sufficient transfection time, whereas 24 h was sufficient for the HCT-116 cell line. Successful transfection was confirmed using both RT-qPCR (Figure 2A) and a Western blot (Figure 2B). Compared to the empty vector, *CARD11* expression was 29,193 and 5043 folds higher in *CARD11* transfected HCT-116 and HT-29 cell lines, respectively (Figure 2A).

In addition, the Western blot showed clear overexpression of *CARD11* compared to control and empty pCDNA3 transfection in both HCT-116 and HT-29 (Figure 2B). The results indicated that there is a correlation between *CARD11* mRNA and protein levels, confirming a similar finding of *CARD11* in clear cell renal cell carcinoma [17].

### 2.2. CARD11 Overexpression Induces NF-κB Activation In Vitro

**Figure 3:**
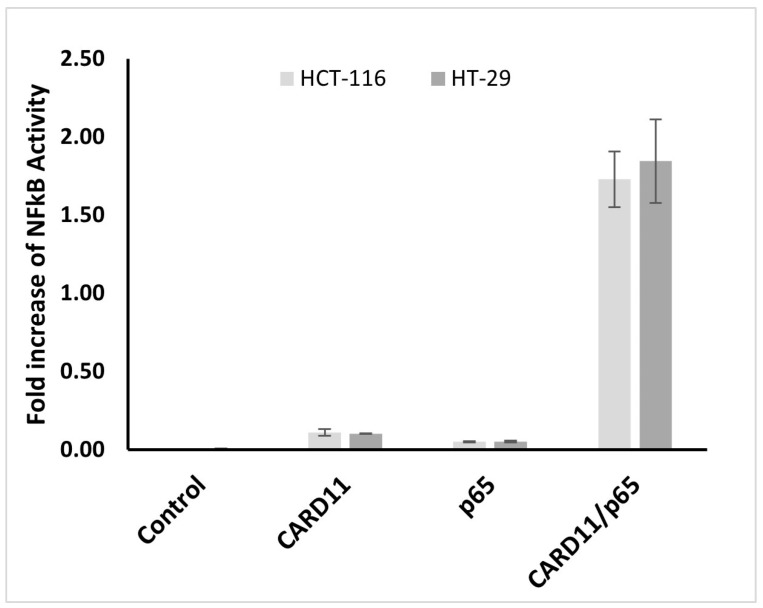
*CARD11* enhances NF-κB activation in both HCT-116 and HT-29 cell lines. The cells were co-transfected with NF-κB-luc vector (with NF-κB luciferase reporter gene-p65) or *CARD11* plasmid alone or together. LPS induction was undertaken for 6 h. NF-κB activation was measured in triplicate experiments and recorded as a fold increase in the vector control.

To determine whether *CARD11* overexpression influences NF-κB, a dual luciferase NF-κB reporter assay was used to test NF-κB activation in *CARD11*-overexpressed HCT-116 and HT-29 CRC cell lines. Results from three biological replicates indicate that endogenous NF-κB activation was not significant in both cell lines. However, the NF-κB pathway significantly increased (*p* < 0.001) with the overexpression of *CARD11* in both cell lines (Figure 3). *CARD11*-mediated activation of NF-κB in HCT-116 and HT-29 cells showed a significant increase of 34.1- and 73.3-fold, respectively.

### 2.3. Overexpression of CARD11 Induces Distinct Transcriptional Profiles in CRC Cell Lines

As the dual luciferase assay (DLA) showed the potential role of *CARD11* in NF-κB activation, we further investigated the effect of *CARD11* overexpression on the transcriptomic level in both cell lines. RNAs extracted from HCT-116 and HT-29, expressing the empty pcDNA3 vector (control) and the *CARD11*-pcDNA3.1 chimera, were subjected to RNA-seq. Principal component analysis (PCA) showed a clear separation between *CARD11* overexpressing and control samples in HCT-116 (Supplementary Materials Figure S1A) and HT-29 cells (Supplementary Materials Figure S1B), confirming the reproducibility of the replicates and the unique transcriptomic profile associated with the overexpression of *CARD11*.

RNA-seq results showed that *CARD11* was significantly differentially expressed between empty vector-transfected (control) and *CARD11*-pcDNA3.1-transfected HCT-116 and HT-29 (Supplementary Materials Figure S2A). This validates the RNA-seq methodology, showing, as expected, that *CARD11* mRNA is overexpressed in *CARD11*-pcDNA3.1-transfected cell lines.

The differential gene expression analysis showed distinct gene expression profiles amongst *CARD11*-transfected HCT-116 and HT-29 cell lines compared to empty vector-transfected cell lines. Following normalization and filtering, a total of 2995 and 3118 differentially expressed genes (DEGs) were found in *CARD11*-transfected HCT-116 and HT-29 cell lines compared to the control, respectively. The DEGs lists resulting from *CARD11* overexpression of HCT-116 and HT-29 are listed in Supplementary Materials Table S1A,B.

**Figure 4:**
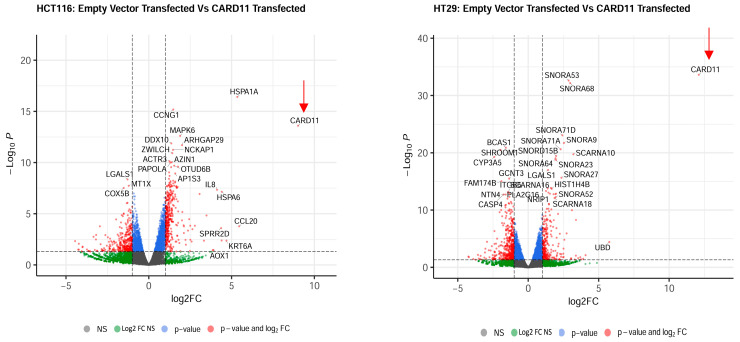
Volcano plots of differentially expressed genes. Genes that are expressed significantly higher in either empty vector- or *CARD11*-transfected cell line based on log2-fold change *p* < 0.05 are highlighted by red dots, *p* > 0.05 are highlighted by green dots (Log2FC NS), unchanged transcripts are demarcated as grey (NS). The red arrows indicate that the *CARD11* expression is significantly upregulated only in the *CARD11*-transfected cell lines.

The most upregulated and downregulated DEGs are also annotated in the volcano plots, and based on log2-fold changes of <−1.5 and >1.5 and *p* < 0.05, 186 and 215 genes were shown to be significantly differentially expressed in *CARD11*-transfected HCT-116 and HT-29 cell lines, respectively (Figure 4).

### 2.4. GSEA of DEGs Revealed Distinctive CARD11-Mediated Activation of the Tumor Immune Microenvironment and Cancer-Related Cellular Pathways

To identify the activated and significantly enriched pathways in each comparison of *CARD11*-transfected vs. empty vector-transfected for both HCT-116 and HT-29, absolute GSEA (absGSEA) was performed on the gene sets (C2, C5, C6, and C7) covering various pathways related to biological processes, and molecular functions, cancer hallmark, and immune response. The number of significantly enriched pathways that derived from absGSEA in *CARD11* overexpressed vs. empty vector for both HCT-116 and HT-29 CRC cell lines are provided in Supplementary Materials Figure S3 as an upset plot graph. It shows there was a significant increase in the overall activation of pathways in HCT-116, particularly those related to immune responses.

A detailed list of the significantly enriched pathways in *CARD11*-transfected vs. empty vector-transfected for both cell lines (Supplementary Materials Table S2A,B), in absGSEA is given in Supplementary Materials Table S2.

**Figure 5:**
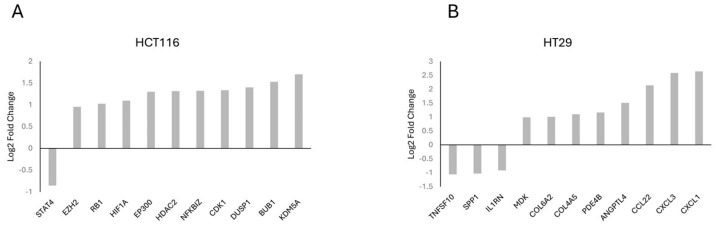
Histogram of the selected leading-edge genes based on frequency in *CARD11*-transfected vs. empty vector-transfected HCT-116 (**A**), HT-29 (**B**) cell lines.

Gene frequency was obtained by counting the number of times a gene occurred across all the different pathways in the cell lines and patients. The genes were obtained from the significant number of enriched genes of the significant gene sets. This suggests that these genes are highly influential because of the overexpression of *CARD11*. The leading-edge genes are represented by the histograms (Figure 5).

The top genes based on the gene frequency and significantly activated cellular pathways between *CARD11*- and empty vector-transfected CRC cell lines showed key genes, including *EP300*, *STAT4*, *RB1,* and *HDAC2* for HCT-116 and *CXCL1*, *CXCL3*, *CCL22,* and *IL1RN* for HT-29.

Among the top enriched genes, three of them (*HIF1A*, *NFKBIZ*, *DUSP1*) for HCT-116 (Figure 5A) and five of them (*CXCL1*, *CCL22*, *IL1RN*, *MDK*, *SPP1*) for HT-29 (Figure 5B) were NF-κB-inducible genes, which is consistent with the dual luciferase assay experiment findings that showed the constitutive activation of NF-κB via *CARD11* expression. Among these five genes in HT-29, three of them (*CXCL1*, *CCL22*, *IL1RN*) were found in the top four of the frequently occurring gene list, indicating that HT-29 probably has more constitutive NF-κB activity than the HCT-116 cell line, again supporting the DLA findings.

A detailed list of selected leading genes based on frequency in *CARD11*- vs. empty vector-transfected cell lines is also provided in Supplementary Materials Table S3A,B as a summary.

### 2.5. Gene Set Enrichment Analysis on CRC Cell Lines Revealed Distinctive CARD11-Mediated Activation of Cellular Pathways Related to Cell Cycle, Apoptosis, Chromatin Remodeling, and Chemotaxis

Having identified frequently occurring genes for each group, significant pathways were selected from the list of pathways obtained from the absGSEA based on *p* < 0.01. Then, given that the key focus of the study was to define the putative role of *CARD11* in CRC pathogenesis, we chose gene sets that contained functional pathways especially linked to B- and T-cell immune responses, cancer hallmarks, and inflammation.

Among the significant pathways, 40 gene sets for the HCT-116 cell line and 23 gene sets for the HT-29 cell line were selected, which were found to be related to B- and T-cell mediated immunity, cancer hallmarks, and inflammation (Supplementary Materials Table S4A,B).

**Figure 6:**
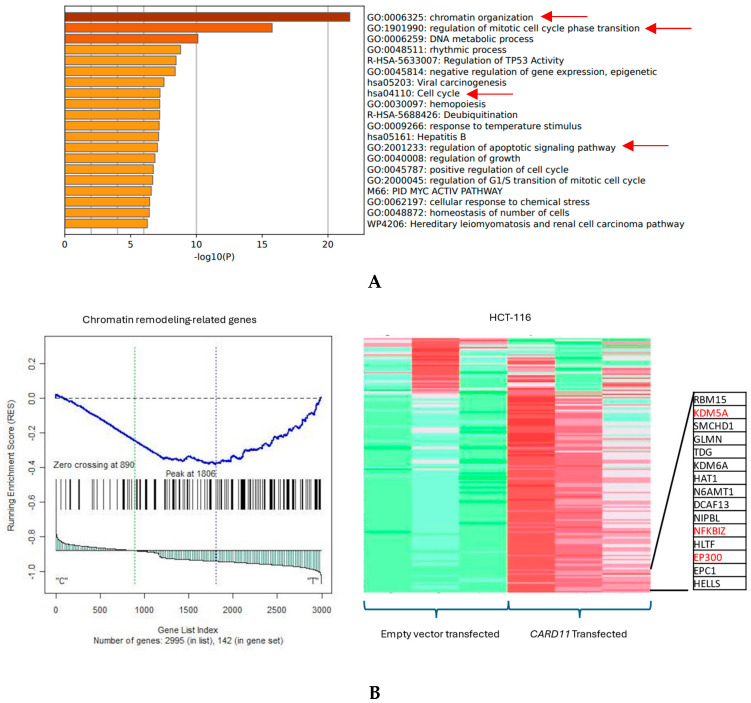
(**A**) Significant enrichment pathways based on frequency in *CARD11*-transfected vs. empty vector-transfected HCT-116 cell line. Red arrows show the interest-enriched pathways in HCT-116. (**B**) Leading edge analysis showed that 82 core genes accounted for the significant enrichment in the *CARD11*-transfected HCT-116 cell line (*p* < 0.001). The top 15 leading-edge core genes are shown; the frequently found ones are indicated in red.

The functional annotation of the top leading-edge genes showed highly significant enrichment of categories related to chromatin organization, cell cycle, and regulation of the apoptotic signaling pathway for the HCT-116 cell line, shown with red arrows in Figure 6A. Absolute GSEA results revealed that a subset of chromatin modeling genes was over-represented in the *CARD11*-transfected HCT-116 cell line (*p* < 0.001, Figure 6B).

**Figure 7:**
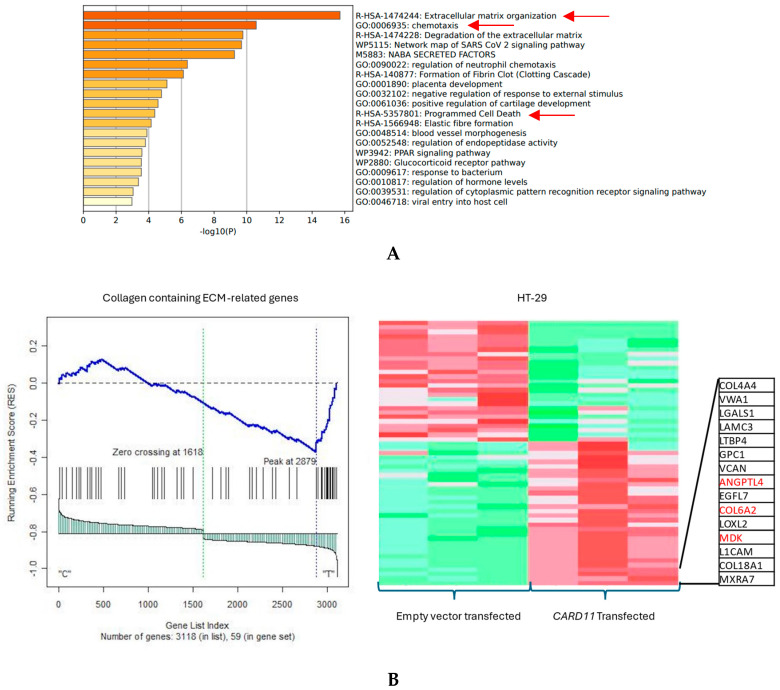
(**A**) Significant enrichment pathways based on frequency in a *CARD11*-transfected vs. empty vector-transfected HT-29 cell line. The red arrows show the significant pathways involved in cancer progression (**B**) Leading edge analysis showed that 20 core genes accounted for the significant enrichment in the *CARD11*-transfected HT-29 cell line (*p* < 0.001). The top 15 leading-edge core genes are shown; the frequently found ones are indicated in red.

The functional annotation of the top leading-edge genes showed significant enrichment of categories related to extracellular matrix organization, chemotaxis, and programmed cell death for the HT-29 cell line (Figure 7A). The expression of a subset of ECM organization genes was over-represented in the *CARD11*-transfected HT-29 cell line, which is a hallmark of cancer (Figure 7B).

Results from the GSEA and Metascape (for functional annotations) showed that *CARD11* is involved in the regulation of cancer-related pathways, including apoptosis, proliferation, cell cycle, and chromatin remodeling in the HCT-116 cell line. However, in the HT-29 cell line, *CARD11* seemed to be more involved with the activation of chemotaxis and extra-cellular matrix (ECM) pathways, which are seen in metastatic cancers, suggesting that perhaps HT-29 is possibly more invasive.

### 2.6. Transcriptional Profiling in CRC Patients Based on CARD11 Differential Expression

We next examined the transcriptional patterns in human formalin-fixed, paraffin-embedded (FFPE) tissue specimens from CRC patients with variable expression levels of *CARD11*, as cell lines exhibit a homogenous system, and a two-dimensional culture might not be a true reflection of an actual tumor mass and its associated tumor microenvironment. To achieve this objective, 10 patients with tubular adenoma and 13 patients with primary CRC were included in the study.

After RNA extraction, whole transcriptome sequencing was conducted for a total of 23 CRC samples. The stratification of patient samples to *CARD11*+ and *CARD11*− was determined by applying a median-centered cutoff (in this study, the median was 73) on the RNA-Seq data. Samples with values below the median cutoff were considered to be *CARD11*−, and those above the median cutoff were considered to be *CARD11*+. According to the RNA-seq data, among 10 adenoma patients, four samples were identified as having low *CARD11* expression (*CARD11*−), and six samples were identified as having high *CARD11* expression (*CARD11*+), whereas in 13 carcinoma samples, eight samples were identified as having low *CARD11* expression (*CARD11*−) and five samples were identified as having high *CARD11* expression (*CARD11*+).

Principal component analysis (PCA) showed a slight admixture between the *CARD11*− and *CARD11*+, mostly likely due to the inherent intra-tumoral heterogeneity for adenoma (Supplementary Materials Figure S1C) and carcinoma (Supplementary Materials Figure S1D) patients’ biopsies. In addition, RNA-seq results showed that *CARD11* had a higher trend in *CARD11*− compared to *CARD11*+ adenoma and was significantly differentially expressed in carcinoma patients (Supplementary Materials Figure S2B).

The differential gene expression analysis showed distinct gene expression profiles between *CARD11*+ patient samples compared to *CARD11*− patient samples. Following normalization and filtering, the DEGs found in adenoma and carcinoma patients for *CARD11*− compared to *CARD11*+ expression were 1132 and 1017, respectively. The DEGs lists resulting from *CARD11* overexpression of adenoma and carcinoma CRC patients are listed in Supplementary Materials Table S1C,D.

**Figure 8:**
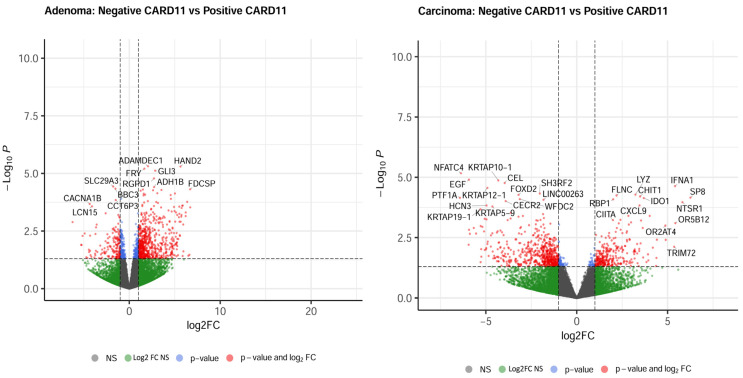
Volcano plots of differentially expressed genes. Genes that were expressed significantly higher in either *CARD11–* and *CARD11*+ patients based on log2 fold change *p* < 0.05 are highlighted by red dots, *p* > 0.05 are highlighted by green dots (Log2FC NS), unchanged transcripts are demarcated as grey (NS).

The most upregulated and downregulated DEGs are also annotated in the volcano plots, and based on log2-fold changes of <−1.5 and >1.5 and *p* < 0.05, 521 and 583 genes were significantly differentially expressed in *CARD11*+ adenoma and carcinoma patients, respectively (Figure 8).

absGSEA was performed to identify the activated and significantly enriched pathways in each comparison of *CARD11*− vs. *CARD11*+ for both adenoma and carcinoma as well. The number of significantly enriched pathways that derived from absGSEA in *CARD11*− vs. *CARD11*+ CRC patients are provided in Supplementary Materials Figure S3 as an upset plot graph. It showed that adenoma has more immune response activated than carcinoma.

A detailed list of the significantly enriched pathways in *CARD11*− vs. *CARD11*+ for both tissue samples (Supplementary Materials Table S2C,D) in absGSEA is given in Supplementary Materials Table S2.

**Figure 9:**
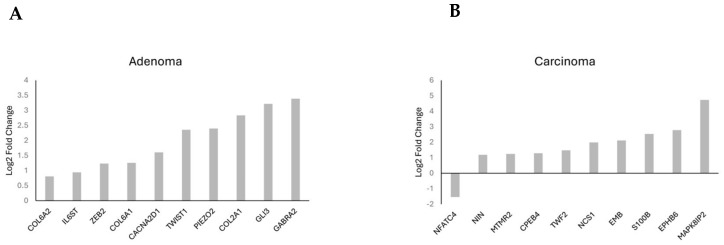
Histogram of the selected leading-edge genes based on frequency in *CARD11*− vs. *CARD11*+ adenoma (**A**) and carcinoma (**B**) patient samples.

Gene frequency was obtained by counting the number of times a gene occurred across all the different pathways in the cell lines and patients. The genes were obtained from the significant number of enriched genes of the significant gene sets. This suggested that these genes are highly influential because of the overexpression of *CARD11*. The leading-edge genes are represented by the histograms (Figure 9).

The detailed list of selected leading genes based on frequency for *CARD11–* vs. *CARD11*+ in adenoma and carcinoma (Supplementary Materials Table S3C,D) is also provided in Supplementary Materials Table S3 as a summary.

### 2.7. Gene Set Enrichment Analysis Revealed Transcriptomic Changes Related to Inflammation, Tumor Immune Microenvironment, and Cancer Hallmark Pathways in CARD11− Compared to CARD11+ Patients

**Figure 10:**
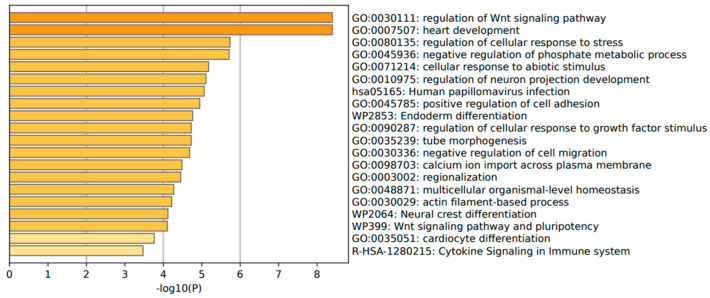
Significant enrichment pathways based on frequency in *CARD11*− vs. *CARD11*+ in adenoma.

The annotation of the top leading-edge genes showed enrichment of categories related to response to abiotic stimulus, immune response (human papillomavirus infection), and cytokine signaling in the immune system for adenoma patients (Figure 10).

Among the significant pathways, 10 gene sets for adenoma and 14 gene sets for carcinoma patient samples were selected, which were found to be related to especially B- and T-cell mediated immunity, cancer hallmarks, and inflammation (Supplementary Materials Table S4C,D).

**Figure 11:**
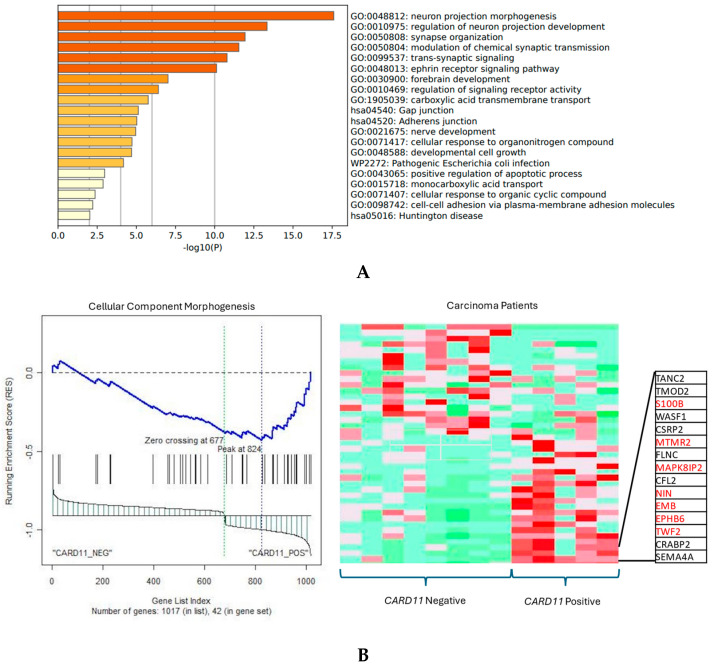
(**A**) Significant enrichment pathways based on frequency in *CARD11*− vs. *CARD11*+ in carcinoma. (**B**) Leading edge analysis showed that there was a significant gene enrichment in the cellular component morphogenesis pathway in *CARD11*-positive carcinoma patients (*p* = 0.0019). The top 15 leading-edge core genes are shown; the frequently found ones are indicated in red.

The functional annotation of the top leading-edge genes showed significant enrichment of categories related to the adherens junction, developmental cell growth, and cellular response to organic cyclic compounds for carcinoma patients (Figure 11A). In addition to promoting and stabilizing cell–cell adhesion, the adhesion junction regulates the actin cytoskeleton, intracellular signaling, and transcription. In carcinoma patients there was a direction of affected pathways related to cellular component morphogenesis and cell cycle, and an adherens junction-enriched pathway found from Metascape also supports these findings. In epithelial cells, the adherens junctions (AJs) are an essential aspect. Their dysregulation is an important step in tumor metastasis because they regulate epithelial tissue architecture and integrity. As a crucial part of cancer progression, AJ remodeling plays a key role in tumor cell growth, survival, and dissemination [18].

### 2.8. Validation of Genes Related to CARD11 Overexpression in CRC

**Figure 12:**
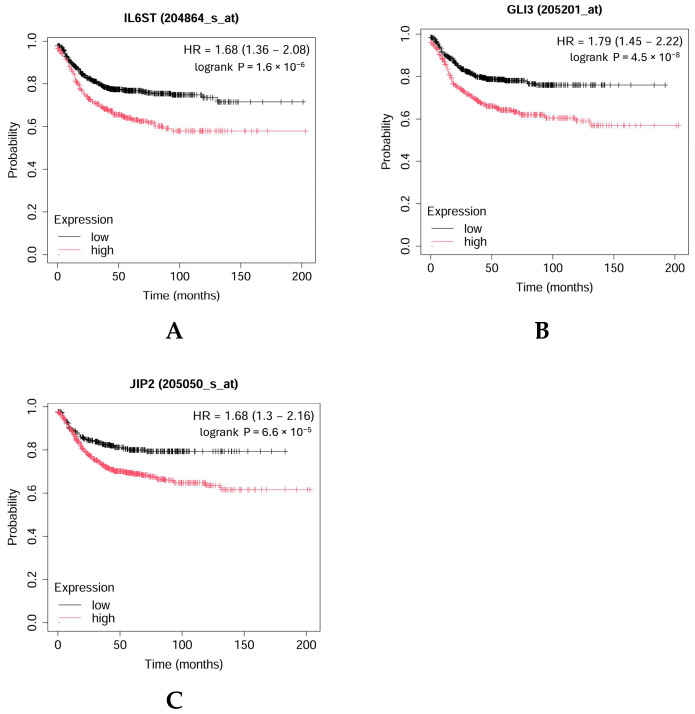
Kaplan–Meier overall survival plot for colorectal cancer patients based on *IL6ST* (**A**), *GLI3* (**B**), and *MAPK8IP2* (*JIP2*) (**C**) expression (https://kmplot.com (accessed on 17 September 2024)).

For the validation of some key genes identified in adenoma (*IL6ST*, *GLI3*) and carcinoma (*MAPK8IP2*, *CPEB4*) patients, survival analysis was completed by using the Kaplan–Meier Plotter (Figure 12A–C). As Figure 12 shows, higher levels of *IL6ST*, *GLI3*, and *MAPK8IP2* were associated with worse overall survival (OS) in CRC patients.

There is a study that showed an increase in CPEB4 protein levels in colorectal cancer (CRC) patient samples using a Western blot analysis (Supplementary Materials Figure S4). The Western blot data provided direct evidence of elevated CPEB4 protein in CRC tissues compared to normal counterparts. In the same study, high *CPEB4* expression was correlated with advanced tumor stage, lymph node metastasis, distant metastasis, and poor prognosis in patients with colorectal cancers [19] (Supplementary Material Figure S4).

### 2.9. Identification of Immune Cell Types in CARD11 Overexpressed CRC Cell Line and Patients

**Figure 13:**
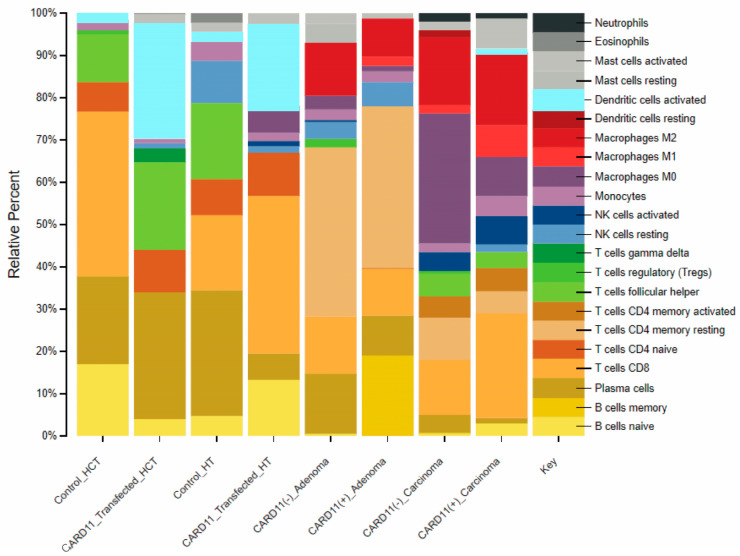
Comparison of CIBERSORTX immune cell fractions between *CARD11*-transfected vs. empty vector-transfected for both cell lines, as well as *CARD11*− versus *CARD11*+ for both tissue samples.

Investigation of the immune cell distribution profile between the cell lines and patients using CIBERSORTX, which was applied to transcriptomic data of CRC cell lines and patients, showed that *CARD11* expression plays a significant role in modulating various types of immune response related to CRC progression and pathogenesis (Figure 13).

For HCT-116, the results showed a 2.57-fold decrease in CD8 T cells and a 1.4-fold increase in the proportion of plasma B cells, while the proportion of naïve B cells showed a decrease in the HCT-116 *CARD11*-transfected cell line, suggesting that plasma B cells may play a role in CRC progression. On the other hand, *CARD11*-transfected HT-29 cells results exhibited a 0.21-fold decrease in plasma B cells and a 2.83-fold increase in naive B cells. Moreover, the proportion of the CD8 T cell fraction increased by two-fold. This suggests a possible interplay between B and T cell immune response in CRC pathogenesis.

In CRC patients, the results showed distinct immune cell types with different proportions between *CARD11*− and *CARD11*+ patients in adenoma and carcinoma. Adenoma patients exhibited higher fractions of CD4 memory resting T cells compared to other groups (with a 0.95-fold decrease in *CARD11*+ compared to *CARD11*− adenoma). Whereas carcinoma cases showed higher fractions of M0-type macrophages (with a 3.33-fold increase in *CARD11*− compared to *CARD11*+ carcinoma) as opposed to other groups. This suggested a more complex interplay between innate and acquired immune response in CRC progression and pathogenesis. The detailed information for the fold changes is provided in Supplementary Materials Table S5.

## 3. Discussion

This study showed that in CRC, the overexpression of *CARD11* leads to dysregulation in NF-kB. This subsequently activates many cellular pathways and genes related to modulation of tumor immune microenvironment (TIME) and cancer-related pathways. This may impact the discovery of putative early diagnostic and prognostic biomarkers and may help identify downstream therapeutic targets for CRC.

As part of this study, two colorectal carcinoma cell lines from different stages of CRC (HCT-116 and HT-29) were used to elucidate some of the molecular mechanisms involved in *CARD11* overexpression and the subsequent activation of cellular pathways related to immune response and cancer progression. HCT-116 is an aggressive colorectal cancer cell line originating from the primary metastatic tumor stage, whereas HT-29 represents a primary tumor in an early stage [16]. This can influence how these cells respond to *CARD11* overexpression and subsequent pathway activation.

Using dual luciferase NF-κB reporter assays, the results showed that the *CARD11* overexpression had an effect on NF-κB activation in both HCT-116 and HT-29 CRC cell lines. *CARD11* overexpression in HT-29 showed higher activation of NF-κB compared to HCT-116. However, for both cell lines, *CARD11* expression had an additive effect on NF-κB activation. Additionally, many of the top ten leading edge genes are NF-κB-inducible genes. Taken together, the results from bioinformatics analysis and the dual luciferase assays suggested that *CARD11* overexpression plays a role in NF-κB activation in CRC.

As the DLA showed the potential role of *CARD11* in NF-κB activation, we further investigated the effect of *CARD11* overexpression on the transcriptomic level in both cell lines. Transcriptomics analysis indicated distinct expression profiles in both cell lines with *CARD11* overexpression. The analysis showed there were more genes upregulated in HCT-116 compared to the HT-29 cell line, indicating that *CARD11* overexpression has a more pronounced effect on the HCT-116. While HT-29 maintained a more stable expression pattern, HCT-116 showed significant activation of pathways related to immune responses.

Functional annotation of the top leading-edge genes in the HCT-116 cell line revealed highly significant enrichment in categories related to chromatin organization, cell cycle regulation, and regulation of apoptotic signaling pathways. Several frequently occurring genes were identified for HCT-116, including *EP300*. *EP300* is a known histone acetyltransferase involved in chromatin remodeling. It has been implicated in various cancers, such as bladder cancer [20], and in CRC, where it interacts with genes such as *BAX* and *BCL2* [21]. This indicates that *EP300* has a role in CRC pathogenesis and patient survival.

*STAT4* has been shown to play a role in tumor progression in CRC. A higher level of *STAT4* expression is associated with increased invasiveness of CRC cells, while inhibition of *STAT4* reduces the growth and invasion [22]. Some key cell cycle genes were also shown to be correlated with *CARD11* overexpression in HCT-116, including *RB1*, *CDK1,* and *EZH2*. 

Interestingly, *CARD11* was co-expressed with IκB-ζ (NFKBIZ), one of the inhibitors in NF-κB signaling. Dysregulation of IκB-ζ disrupts the regulation of the canonical NF-κB pathway, leading to abnormal activation of various NF-κB target genes, including MAPK, E2F, and CDK1, among others. This suggests that modifications in the IκB-ζ expression may contribute to the improper control of cell cycle and survival pathways in CRC. Further exploration of how *CARD11* influences this process is necessary to fully understand the implications of CRC.

Additionally, a few genes, such as *HDAC2* and *KDM5A*, related to chromatin remodeling were co-expressed with *CARD11*. *KDM5A* facilitates the recruitment of chromatin remodeling complexes to specific genomic loci, enabling gene expression changes in response to various cellular signals, including DNA damage [23]. In addition, *KDM5A* also regulates B cell proliferation and differentiation, essential for developing antibody-secreting plasma cells and memory B cells. Studies have shown that *KDM5A* can influence B cell differentiation in germinal centers, affecting specific IgG antibody production in response to antigens [24], which may explain the increase in plasma B cells observed in the CIBERSORTX analysis.

The functional annotation of the top leading-edge genes showed significant enrichment of categories related to extracellular matrix organization, chemotaxis, and programmed cell death for the HT-29 cell line. As solid cancers progress, the ECM undergoes significant changes in composition and function, which enable cancer cells to grow and spread. In most tumor tissues, the ECM remodeling is characterized by increased collagen synthesis, which explains why we find more collagen-related genes in frequency analysis [25].

The top two frequently occurring genes in the *CARD11*-transfected HT-29 cell line were *CXCL1* and *CXCL3* chemokines. *CXCL1* promotes tumor progression by enhancing cell growth, motility, invasion, angiogenesis, and metastasis, making it a critical factor in the aggressive behavior of cancer cells across different types of cancer. *CXCL1* has been implicated in various cancers, including triple-negative breast cancer [26]. It is also shown that *CXCL1* promotes colon cancer development through the activation of NF-κB/P300 [27]. *CXCL3* has also been linked to attracting natural killer (NK) cells, T helper 1 (Th1) cells, monocytes, and CD8+ T cells, which are crucial in the immune response against tumors, which confirms our findings [28]. *CCL22* was the other frequently occurring gene we identified in the HT-29 cell line. Both *CXCL1* and *CXCL3* genes, along with *CCL22*, are involved in the chemotaxis of tumor cells and stromal cells within the surrounding microenvironment, which is essential in tumor dissemination during progression and metastasis. *CCL22* has been shown to regulate chemotaxis and to be involved in the recruitment of T cells and other immune cells in breast, ovarian, and gastric cancers and leukemia [29].

As cell lines exhibit a homogenous system, and a two-dimensional culture might not be a true reflection of an actual tumor mass and tumor microenvironment, we next examined the transcriptional patterns in human FFPE tissue specimens from CRC patients with variable expression levels of *CARD11*. For patients, *CARD11* overexpression was associated with different transcriptomics profiles at various stages of CRC. 

Adenoma patients showed enrichment in pathways related to inflammation, immune response, and TIME. One gene co-expressed with *CARD11* was the IL-6 Receptor Subunit Beta (*IL6ST*), associated with IL6. Interestingly, IL6 modulates the TIME to facilitate metastatic colonization of colorectal cancer cells [30]. Moreover, *IL6ST* affects the JAK/STAT pathway in CRC [31]. Other frequently enriched genes in adenoma include collagen family members such as *COL6A1*, *COL6A2*, and *COL6A3*. These genes play crucial roles in cancer development and prognosis through their interactions with the tumor microenvironment and immune responses. Previous studies have shown that collagen proteins are increased in CRC patients [32]. Other genes related to the CRC progression through invasion, such as *GLI3* [33], and proliferation, such as *PIEZO2* [34], were also identified.

CRC carcinoma patients predominantly showed genes related to cancer hallmark pathways. *MAPK8IP2* is associated with the MAP kinase pathway, a common cancer pathway, and encodes the JNK interacting protein 2 or JIP2. In CRC carcinoma patients in this study, it promoted tumor progression, similar to its role in prostate cancer [35]. The MAP kinase pathway can be activated through NF-kB dysregulation in some cancers [36], suggesting a similar interplay in CRC. *MTMR2* promotes invasion and metastasis of gastric cancer via inactivating IFNγ/STAT1 signaling [37] and modulates TIME by promoting the progression of NK/T cell lymphoma by targeting JAK1 [38]. *EPHB6* is an ephrin-B receptor, where its overexpression promotes CRC and modulates the tumor immune microenvironment of primary colorectal adenocarcinomas metastasizing to the liver or lungs [39]. *CPEB4* is involved in the regulation of intestinal inflammation resolution and colorectal cancer development. *CPEB4* is overexpressed in inflammatory cells in patients with IBD and in CRC, favoring tumor development [40]. *S100B* is involved in the regulation of cell cycle progression and differentiation. It was found to be overexpressed in the liver metastases of colorectal cancer patients [41].

In silico validation of overall survival with the key genes and the literature findings support our data from the patients. The IL6ST is a key component in the IL-6/STAT3 signaling pathway, which is known to promote CRC malignancy. Continuous activation of STAT3 by IL-6 signaling is linked to aggressive tumor behavior and poor patient prognosis in CRC [42]. Based on our findings, aside from the IL6ST gene, CRC transition markers, such as GLI3, are also expressed at elevated levels. Studies utilizing data from The Cancer Genome Atlas (TCGA) and Gene Expression Omnibus (GEO) have shown that patients with elevated GLI3 expression experience poorer survival outcomes compared to those with lower levels of GLI3 [33].

MAPK8IP2 is associated with the MAP kinase pathway, a common cancer pathway, and encodes the JNK interacting protein 2 or JIP2. Our study suggested that it also promotes tumor progression, similar to its role in prostate cancer [35]; however, its effects have not been investigated previously in patients with CRC. Kaplan–Meier’s overall survival plot for CRC patients based on MAPK8IP2 expression revealed a significant decrease (*p* < 0.001) in survival for colorectal patients with a higher expression of MAPK8IP2. Studies indicate that the suppression of CPEB4 expression can enhance apoptosis and decrease cellular proliferation in colon cancer cells. This suggests that CPEB4 may help maintain the survival and proliferative capacity of metastatic CRC cells, thereby contributing to their invasive potential. In one of the studies, high CPEB4 expression was correlated with advanced tumor stage, lymph node metastasis, distant metastasis, and poor prognosis in patients with colorectal cancers [19]. It has also been shown that CPEB4 is highly expressed in the peripheral blood of colorectal cancer patients as well [43].

Investigation of the immune cell distribution profile between the cell lines and patients was performed. This was achieved by applying CIBERSORTX to the transcriptomic data. The results identified differences in immune cell types in *CARD11* overexpressed HCT-116 compared to the empty vector, suggesting modulation of TIME related to the progression of CRC. The analysis showed that B cell infiltration of primary CRC was characterized by an accumulation of terminally differentiated memory B cells or plasma cells. This suggested a specific immune response against the tumor [44], most likely through the dysregulation of NF-κB as the NF-κB pathway is essential for generating a complete and diverse B cell pool, with different B cell subsets showing varying degrees of dependence on NF-κB signaling for survival and development [45]. In addition, CD8 T cells were depleted with *CARD11* overexpression, which can be a sign of immune suppression and may lead to cancer progression. 

In the current study, the results showed an increase in the proportion of the NK resting cells in *CARD11*-transfected HCT-116 compared to the control. One of the studies deconvolving immune cells from bulk transcriptomes of 521 human CRCs found that NK cells are present in colorectal tumors, but most adopt a “resting”, non-activated state, which supports our finding. It must be noted that the increase in the resting NK cells of the HT-29 cell line is not significant, which implies that the HT-29 cell line is still capable of balancing itself, unlike the HCT-116 cell line, which became increasingly aggressive and uncontrollable as a result of *CARD11* transfection [46].

CIBERSORTX analysis for the HT-29 cell line showed that *CARD11* overexpression led to a significant increase in CD8 T cells, indicating that the cell line is still intact and attempting to combat cancer cells by infiltrating CD8 T cells to the TIME. The presence of elevated CD8+ T cells in the tumor microenvironment has been linked to a favorable prognosis in cancer [47]. GSEA results for HT-29 revealed enrichment in genes involved in the upregulation of transcripts related to T cell function, consistent with the increased number of CD8 T cells identified in this study. However, in the HCT-116 control, a fraction of CD8 T cells already existed but was depleted in *CARD11* overexpressed HCT-116. This demonstrates that the two CRC cell lines were affected by *CARD11* overexpression in different ways.

In CRC patients, the results showed distinct immune cell types with different proportions between *CARD11–* and *CARD11*+ patients in adenoma and carcinoma. Adenoma patients exhibited higher fractions of CD4 memory resting T cells compared to other groups., whereas carcinoma cases showed higher fractions of M0-type macrophages as opposed to other groups. This suggested a more complex interplay between innate and acquired immune responses in CRC progression and pathogenesis. Moreover, there seemed to be a difference in the distribution profile of immune cells between different cell lines and patients, supporting the conclusion in this study that obtaining different GSEA results is as expected.

There are a few limitations to this study. Whilst the use of CIBERSORT to predict the immune cell environment is based on in silico analysis, it provides insights into the possible behavior of the tumor cells to secrete or behave like immune cells through their transcriptome profile. In addition, it is worth noting that the transcriptomic analysis in CRC cell lines was conducted post-transient transfection in HCT-116 and HT-29. It is possible that short-term transfection in CRC cell lines will result in transcriptomic changes that are distinct from those observed in cell lines evolved under selective pressure for *CARD11* overexpression. The results from this study warrant further work to investigate some of the molecular mechanisms of *CARD11* in CRC. Such studies can be conducted using 3D organoid models. In spite of these limitations, the following two points address such limitations to a large extent: (a) the analysis involved *CARD11–* and *CARD11*+ CRC patient samples, which is a better in vivo model with more intact TIME than the homogenous CRC cell line model, 3D organoid model, or the in vivo mouse model; and (b) two cell lines from different stages of CRC were used in this study to investigate the effect of *CARD11* overexpression on CRC.

Despite the inherent limitations of the current study due to the relatively limited sample size and lack of validation in a broader population, the results indicate that transcriptional profiling can be a valuable method for identifying biomarkers that are involved in the pathogenesis of *CARD11*+ and *CARD11–* CRC. It is imperative that additional research be conducted across different population cohorts to better understand how *CARD11* may play a role in colorectal cancer initiation and progression.

Taken together, this study showed the involvement of *CARD11* overexpression in CRC pathogenesis via the dysregulation of the NF-κB pathway. The loss of tight control of the NF-κB pathway leads to a shift in the cellular response related to cell cycle, proliferation, and apoptosis, which are key pathways in CRC as well as modulation in the tumor immune microenvironment associated with CRC. This is indicated by the presence of different types of immune responses in different stages of CRC.

## 4. Materials and Methods

**Figure 14:**
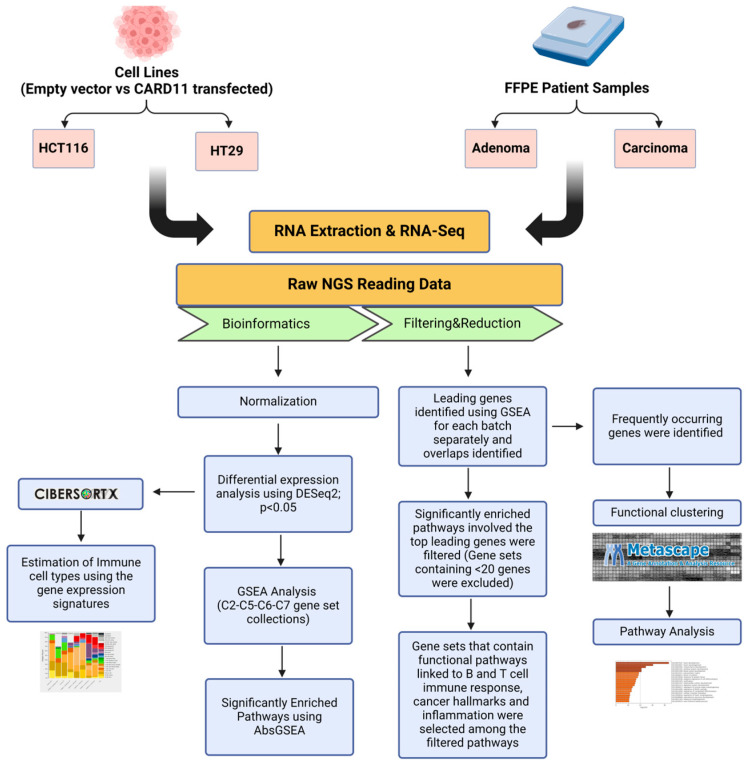
Flowchart outlining the steps of the bioinformatics approach used to identify differentially expressed genes in *CARD11*-transfected to empty vector-transfected cell lines and *CARD11–* to *CARD11*+ in patient samples. The figure was created using BioRender.com (accessed on 17 September 2024).

The schematic diagram of the research methodology is given in Figure 14.

### 4.1. Cell Culture 

This study utilized two different CRC cell lines, HCT-116 and HT-29, to represent different levels of aggressiveness. They were maintained in RPMI 1640 supplemented with 10% fetal bovine serum (Sigma Aldrich, St. Louis, MO, USA) and 1% Penicillin/Streptomycin (Sigma) at 37 °C in a 5% CO_2_ incubator. 

### 4.2. Transfection of CRC Cell Lines with CARD11

Overexpression was performed using *CARD11* cloned in pcDNA3.1 vector (OHu21225D) (GenScript, Piscataway, NJ, USA). Cells were seeded at 2 × 10^5^ cells per well in 6-well plates on the day before transfection. The DNA to use and the incubation time for optimal transfection were optimized for each cell line before the experiments were conducted. The HCT-116 and HT-29 cell lines were transfected with 1 and 2 µg of *CARD11* plasmid construct using ViaFect transfection reagent (Thermo Fisher Scientific, Waltham, MA, USA), according to the manufacturer’s instructions, respectively. To compare the transfection efficiency, the cells of a 6-well plate were kept non-treated as a control, and the cells of a 6-well plate were treated only with ViaFect transfection reagent without adding the DNA as a mock. Cells transfected with the empty pcDNA3.1 vector served as the experimental control. The *CARD11* expression level was checked at 24 and 48 h post-transfection at the mRNA and protein levels using RT-qPCR and a Western blot, respectively.

### 4.3. RNA Extraction

Following 24 and 48 h of transfection, transfected and non-transfected cells were collected by trypsinization to determine the optimal transfection efficiency with *CARD11* using qPCR and Western blot analysis. Cells were pelleted, and RNA was extracted from the cell pellet using the RNeasy kit (Qiagen, Hilden, Germany), according to the manufacturer’s instructions. 

Genomic DNA removal from all RNA extractions was carried out by treating the extracted RNA with a TURBO DNAase-free TM Kit (Invitrogen, Carlsbad, CA, USA).

### 4.4. Quantitative Reverse Transcriptase-PCR (qRT-PCR)

Complementary DNA (cDNA) was synthesized from total RNA using a High-Capacity cDNA Reverse Transcription Kit (Applied Biosystems, Waltham, MA, USA), that uses a mixture of random hexamer and oligo Dt, according to the manufacturer’s instructions. Gene expression was determined by quantitative PCR (qPCR) using Maxima SYBR Green/ROX qPCR MasterMix (2×) (Thermo Fisher Scientific) on QuantStudio3 Real-Time PCR thermal cycler (Applied Biosystems). qPCR runs were performed using the primer sets in Table 1.

The housekeeping gene (18S) was used to normalize the *CARD11* gene expression levels, and the fold change, which measures relative expression, and was calculated using the Comparative Ct (2^–ΔΔCt^) method.

### 4.5. Cell Lysis and Western Blot

For Western blot analysis, after the transfection, the cells were collected and lysed with M-PER Mammalian Protein Extraction Reagent (Thermo Fisher Scientific), supplemented with a protease inhibitor cocktail (Sigma) and DTT (Sigma). Each sample was loaded as 50 ug/well, separated by 10% SDS polyacrylamide gel electrophoresis, and transferred to a 0.45-mm nitrocellulose membrane (Thermo Fisher Scientific). The membrane was incubated with Anti-*CARD11* (CARMA1) (Rabbit monoclonal, dilution 1:1000, ab124730, Abcam, Cambridge, UK) and developed using anti-rabbit IgG as a secondary antibody. β-actin antibody was used as an internal control.

### 4.6. Investigating the Effect of CARD11 Overexpression on NF-κB Activation Using Dual Luciferase Assay

The potential role of NF-κB activation via *CARD11* was analyzed in vitro using a Luciferase Reporter Assay System (Promega, Southampton, UK) in HCT-116 and HT-29 cell lines. Briefly, cells were seeded at 5 × 10^4^ cells per well in 24-well plates. Four different groups were used: an untransfected cell line as a control, transfected with *CARD11* expression vector, transfected with pNF-κB-luc (a Firefly luciferase reporter for NF-κB activity-p65), and the last group was transfected with both *CARD11* and pNF-κB-luc (p65) vectors to see the inducing effect of *CARD11* on NF-κB activity using Viafect reagent (Thermo Fisher Scientific), according to the manufacturer’s instructions, respectively. All the cells in different groups were transfected with pRL-TK (a Renilla luciferase reporter as a control) serving as the internal control.

The cells were harvested, washed with PBS, lysed, and assayed for luciferase activities using a GloMax Plate Reader (Promega), following the manufacturer’s instructions.

### 4.7. Whole Transcriptome Sequencing

A total of 30 ng of extracted and Turbo DNase treated RNAs from HCT-116 and HT-29 cell lines were used to sequence the whole transcriptome using a targeted AmpliSeq Transcriptome Panel, which is designed to target over 21,000 specific human RNA transcripts using a high-throughput multiplexed method on Ion S5 XL Semiconductor Sequencer (Thermo Fisher Scientific, USA) with the Ion 540 Chip (Life Technologies, Carlsbad, CA, USA). In brief, total RNAs were first processed with the SuperScript VILO cDNA synthesis kit (Invitrogen; 11754050), and the resulting cDNAs were amplified using Ion AmpliSeq™ transcriptome human gene expression kit (Thermo Fisher Scientific; A26325) to prepare the libraries. The prepared libraries were purified using Agencourt AMPure XP Beads (Beckman Coulter, Indianapolis, IN, USA), and the purified libraries were quantified using an Ion Library TaqMan™ Quantitation Kit (Applied Biosystems, Waltham, MA, USA). The libraries were diluted to ~100 pM and pooled by combining an equal volume of each barcoded library, then they were amplified using emulsion PCR on Ion OneTouch™ 2 instrument (OT2) (Thermo Fisher Scientific) and enriched on Ion OneTouch™ ES as per the manufacturer’s instructions. Thus, prepared template libraries were then sequenced with Ion S5 XL Semiconductor sequencer (Thermo Fisher Scientific) using the Ion 540™ Chip.

### 4.8. RNA-Seq Data Analysis

RNA-seq data analysis was performed using the Ion Torrent Software Suite version 5.4. To align the raw sequencing reads generated by Ion Torrent sequencing against the reference sequence from the hg19 (GRCh37) assembly, the Torrent Mapping Alignment Program (TMAP) was utilized, and the specificity and sensitivity were maintained by implementing a two-stage mapping approach by employing BWA-short, BWA-long, SSAHA [48], Super-Maximal Exact Matching [49], and the Smith–Waterman algorithm [50] for optimal mapping. The raw read counts of the targeted genes were normalized using the Fragments Per Kilobase Million (FPKM) normalization method.

Principal component analysis (PCA) was applied to the samples using the R statistical software (version 4.2.0). DEG analysis was performed using the DESeq2 R/Bioconductor package with raw read counts from the RNA sequencing data to identify the DEGs in each comparison of cell lines with *CARD11*-transfected against cell lines with empty vector-transfected [51,52].

Genes with fewer than 10 read counts were excluded from further analysis. *p* < 0.05 was used as a level of significance for selecting DEGs close to noise, which is typical of inflammation and immune response genes. DEGs were subjected to GSEA for further downstream filtering and selection of genes based on biological pathways.

### 4.9. Gene Set Enrichment Analysis

The significant DEGs lists obtained were further analyzed to identify the activated and enriched cellular pathways in response to *CARD11* overexpression in comparison to empty vector-transfected cell lines using the absGSEA, as previously described [36]. Absolute GSEA was performed on expression data using around 90,000 annotated cellular pathways obtained from the Broad Institute’s database (https://www.gsea-msigdb.org (accessed on 17 September 2024)) and custom pathways.

For significantly activated pathways, a threshold of *p* < 0.05 was used. Immunity and inflammation pathways were further explored to identify the differentially-enriched genes between the *CARD11*-transfected and empty vector-transfected samples by performing leading-edge analysis as previously described [36]. The resulting gene sets were further reduced by carrying out a systematic cross-reference of each gene enriched within statistically significant pathways. Finally, genes that are highly frequent across multiple significant pathways enriched between the *CARD11*-transfected and empty vector-transfected samples were identified.

### 4.10. Functional Enrichment Analysis by Metascape

Functional clustering and pathway analysis of the common DEGs or frequently occurring genes were performed using Metascape (http://metascape.org (accessed on 17 September 2024)) [53]. To validate the pathways identified using GSEA, the frequently occurring genes across all gene sets were subjected to Metascape analysis.

### 4.11. FFPE Tissue Specimens from Endoscopic Biopsies of CRC Patients

This study included 10 patients with tubular adenoma and 13 patients with primary CRC from the American Hospital Dubai and the University Hospital Sharjah (Table 2). The ethical approval for the study was obtained from the Dubai Scientific Research Ethics Committee (DSREC), Dubai Health Authority (DSREC-SR-02/2023_07), and the Research and Ethics Committee (REC) of the University Hospital Sharjah (UHS-HERC-055-25022019), respectively. The methods were conducted based on the respected guidelines of the Declaration of Helsinki and the Belmont Report. The primary diagnosis was performed to determine and score tumors, lymph nodes, and metastasis (TNM) under the supervision of two pathologists (K.S and R.H).

### 4.12. Validation of Genes Related to CARD11 Overexpression in CRC

Survival analysis was completed by using the Kaplan–Meier Plotter (kmplot.com/analysis/ (accessed on 17 September 2024)) to create the overall survival (OS) curves for some of the key genes identified after GSEA analysis of *CARD11–* and *CARD11*+ samples for both adenoma and carcinoma. *IL6ST*, *GLI3* genes for adenoma and *MAPK8IP2*, *CPEB4* genes for carcinoma were included for the analysis. 

### 4.13. Exploration of Immune Cell Characteristics

Cell-Type Identification by Estimating Relative Subsets of RNA Transcripts (CIBERSORTX) (https://cibersort.stanford.edu (accessed on 17 September 2024)), created by Newman et al., is a deconvolution algorithm based on RNA-seq data that allows for accurate quantification of the relative levels of immune cells classified according to their types in a complex gene expression mixture. The CIBERSORTX algorithm determines a *p*-value that indicates the statistical significance of the deconvolution results across all cell subsets and indicates the degree of confidence in the results [18]. It uses gene expression signatures consisting of approximately 500 genes. We applied the original CIBERSORTX gene signature file termed LM22, which contains 547 genes and distinguishes 22 human hematopoietic cell phenotypes, including 7 T cell types, naïve and memory B cells, plasma cells, NK cells, and myeloid subsets. Normalized gene expression data of the *CARD11*-transfected and empty vector-transfected samples for each cell line and the *CARD11*– and *CARD11*+ samples for both adenoma and carcinoma were uploaded to the CIBERSORTX web portal.

## 5. Conclusions

The study identified genes and pathways related to *CARD11* overexpression in colorectal cancer in both colorectal cancer cell lines and patients. Comparison of whole transcriptome RNAseq analysis showed that *CARD11* appears to play a key role in CRC progression through dysregulation of the NF-κB pathway, promoting cell growth and tissue remodeling in the HCT-116 CRC cell line and inducing chemotaxis and ECM organization pathways in the HT-29 CRC cell line. In the HCT-116 cell line, key genes involved in these pathways included *EP300*, *KDM5A*, *HIF1A*, *NFKBIZ*, and *DUSP1*, as well as *IL1RN*, *MDK*, *SPP1*, and various chemokines, including *CXCL1*, *CXCL3*, *CCL22* in HT-29 cell lines. Genes related to *CARD11* overexpression in colorectal adenoma patients included *IL6ST*, *GLI3*, *and PIEZO2,* as well as the collagen-related gene family, whilst *MAPK8IP2* gene expression was dramatically elevated in *CARD11*+ carcinoma patients. Additional genes upregulated in carcinoma patients as a result of *CARD11* overexpression included *MTMR2*, *EMB*, *EPHB6*, and *CPEB4,* which are related to various cancer-related processes. Taken together, the results showed that *CARD11* overexpression contributes to the progression of CRC through modulation of various tumor immune microenvironment pathways and activation of cancer pathways via the dysregulation of NF-κB. The genes and pathways implicated in *CARD11* overexpression may provide insights into the early diagnosis and potential therapeutic targets for colorectal cancer.

## Figures and Tables

**Table 1:** Sequence of primer pairs used in the qPCR.

Gene ID	Forward Primer Sequence	Reverse Primer Sequence
*CARD11*	AGCGGGACAGCTACAATGAC	TGACCGCCATGTTCTC
18S	TGACTCAACACGGGAAACC	TCGCTCCACCAACTAAGAAC

**Table 2:** Patient characteristics for the 23 biopsies collected from adenoma and adenocarcinoma patients in the UAE.

No	Gender	Age	Nationality	Subtype
1	Male	61	Italian	Tubular Adenoma
2	Male	61	Qatari	Tubular Adenoma
3	Male	54	Emirati	Tubular Adenoma
4	Male	61	Emirati	Tubular Adenoma
5	Female	39	Italian	Tubular Adenoma
6	Male	75	Indian	Tubular Adenoma
7	Female	64	British	Tubular Adenoma
8	Male	48	Portuguese	Tubular Adenoma
9	Female	51	Emirati	Tubular Adenoma
10	Male	50	South African	Tubular Adenoma
11	Female	77	Emirati	Adenocarcinoma
12	Female	83	Syrian	Adenocarcinoma
13	Female	45	Emirati	Adenocarcinoma
14	Male	92	Emirati	Adenocarcinoma
15	NA	80	Iraqi	Adenocarcinoma
16	Female	53	Slovakia	Adenocarcinoma
17	Female	52	Emirati	Adenocarcinoma
18	Male	69	Emirati	Adenocarcinoma
19	Male	75	Sudanese	Adenocarcinoma
20	Female	68	Emirati	Adenocarcinoma
21	Male	76	Emirati	Adenocarcinoma
22	Female	70	Emirati	Adenocarcinoma
23	Female	80	Lebanese	Adenocarcinoma

## Data Availability

The RNA sequencing data generated in this work have been deposited in the Gene Expression Omnibus (https://www.ncbi.nlm.nih.gov/geo (accessed on 17 September 2024)) under GEO Series access number GSE266390 and can be accessed from https://www.ncbi.nlm.nih.gov/geo/query/acc.cgi?acc=GSE266390 (accessed on 17 September 2024). All other supporting data of this study are either included in the manuscript or available on request from the corresponding author.

## Supplementary Materials

The following supporting information can be downloaded at: https://www.mdpi.com/article/10.3390/ijms251910367/s1.
