# Supplementary figures and images for: Transcriptome Profiling Associated with CARD11 Overexpression in Colorectal Cancer Implicates a Potential Role for Tumor Immune Microenvironment and Cancer Pathways Modulation via NF-κB
Source: Int J Mol Sci. 2024 Sep 26;25(19):10367. doi: 10.3390/ijms251910367 (PMC11476988; doi:10.3390/ijms251910367)

**A**

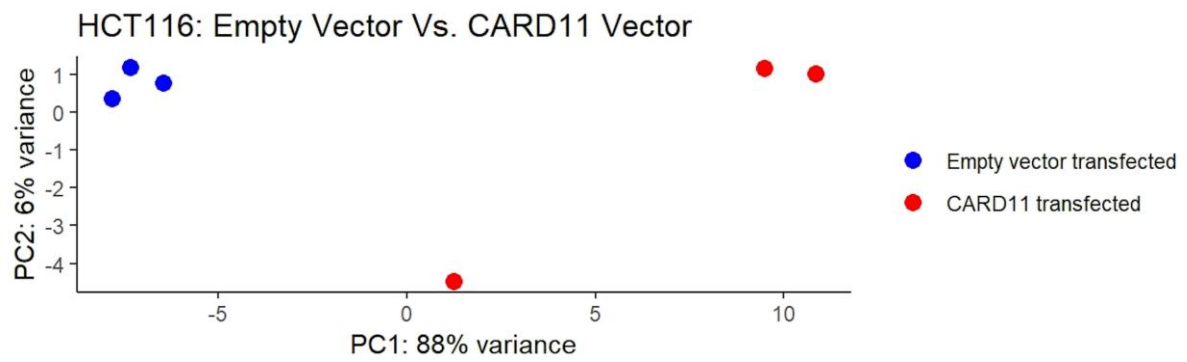

**B**

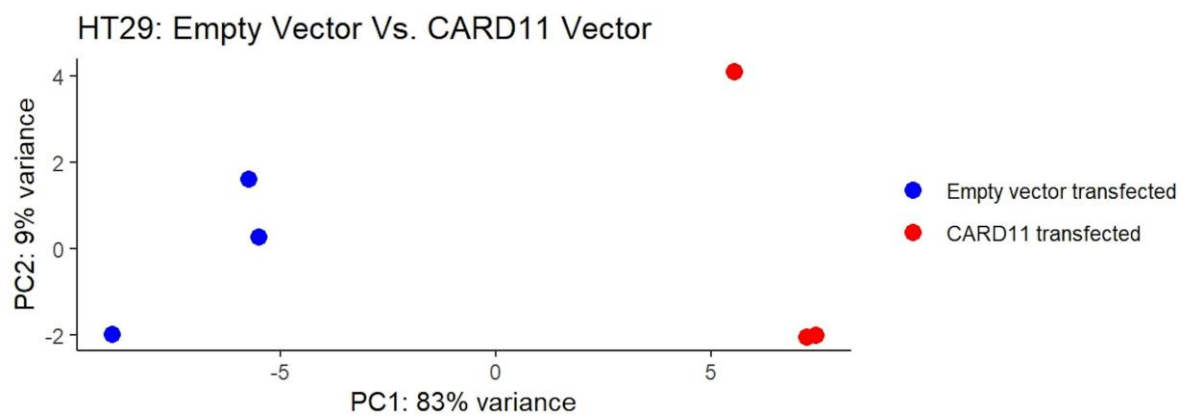

C

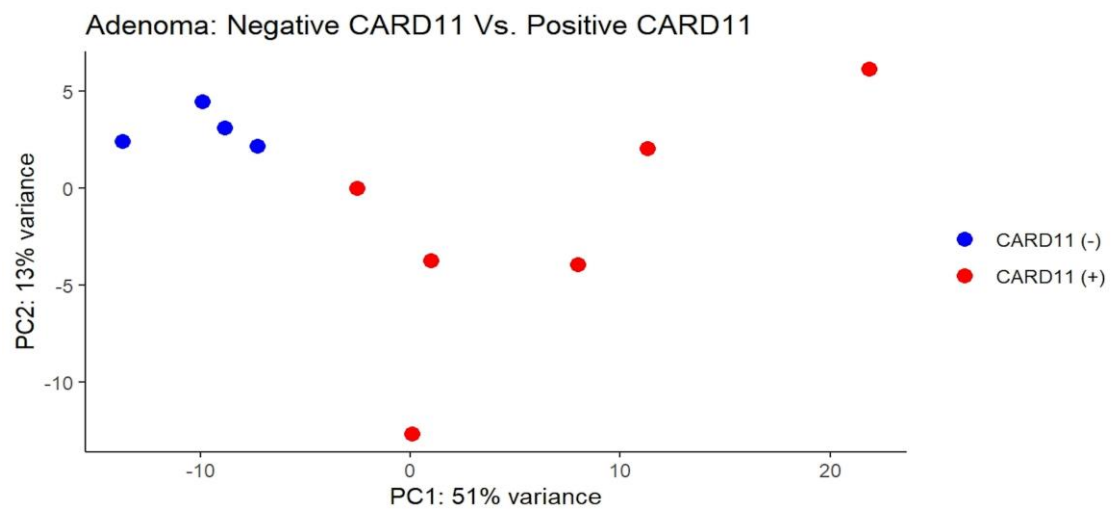

D

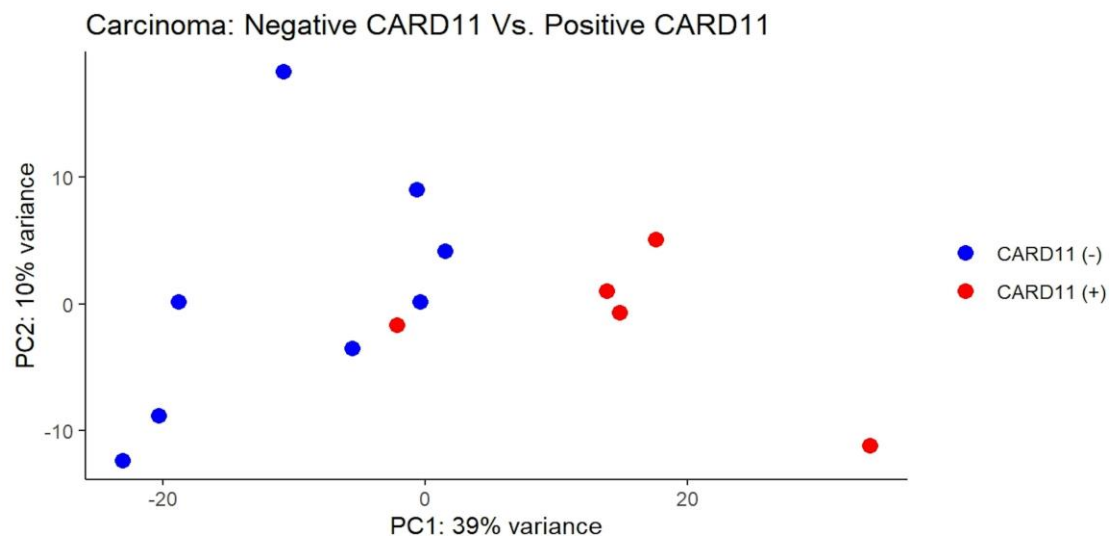

Supplement: Supplementary file 1 [file ijms-25-10367-s001.zip › Figure S1_Principle Component Analysis (PCA)_Cell_Lines_Patients.pdf]

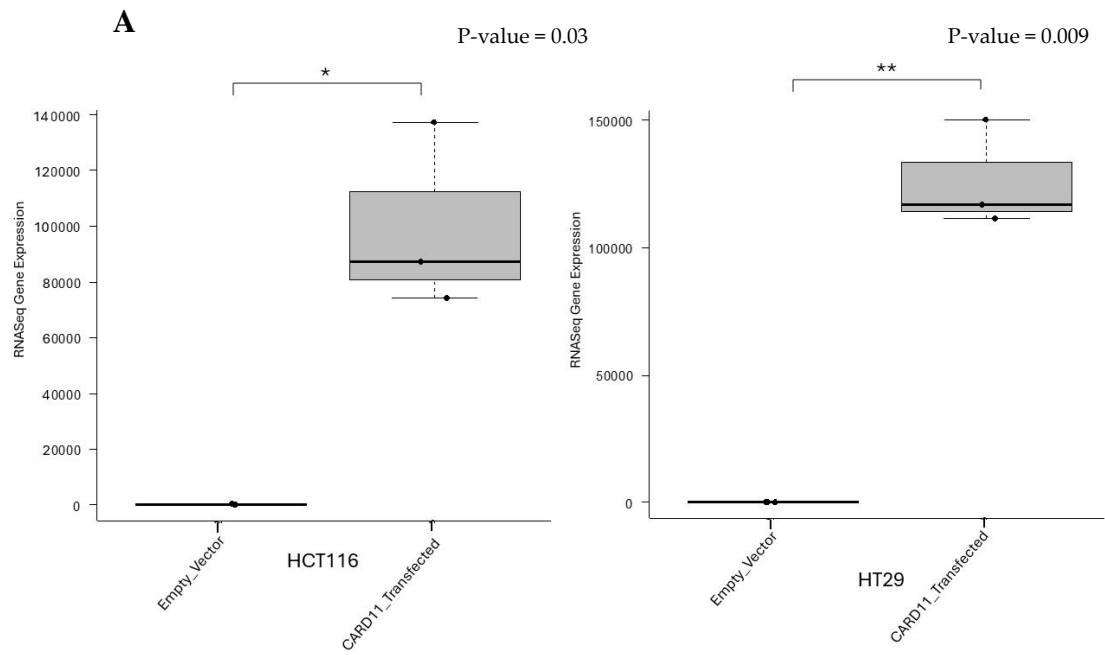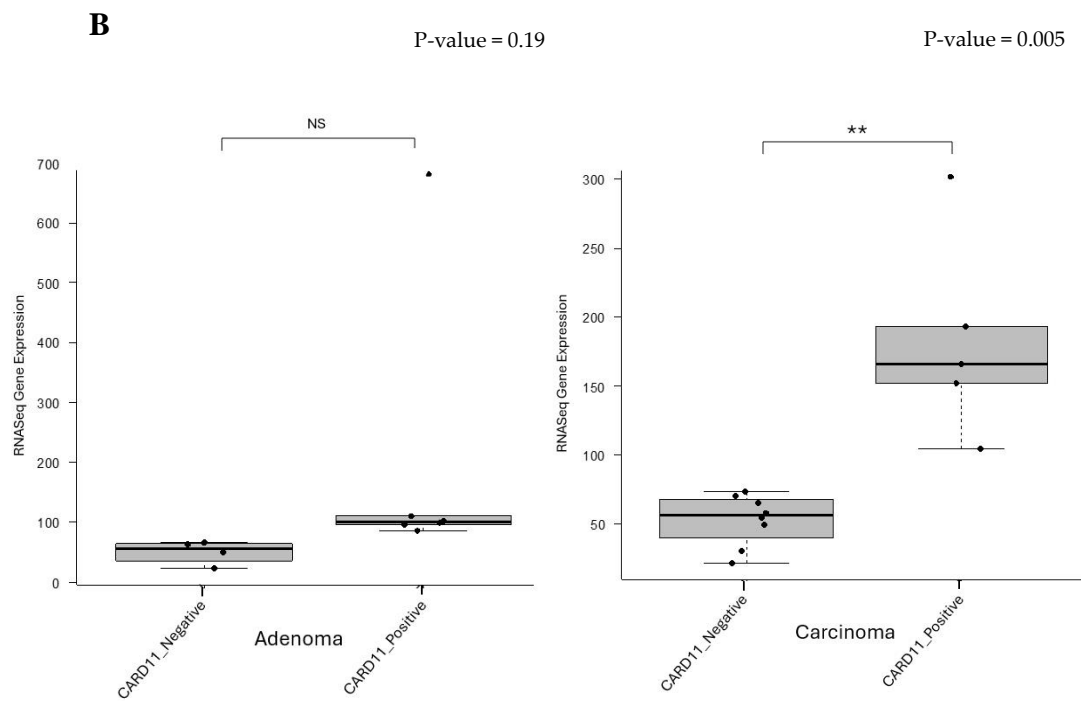

Supplement: Supplementary file 1 [file ijms-25-10367-s001.zip › Figure S2_Boxplots with data points for the expression of CARD11 gene in each cell lines and patients.pdf]

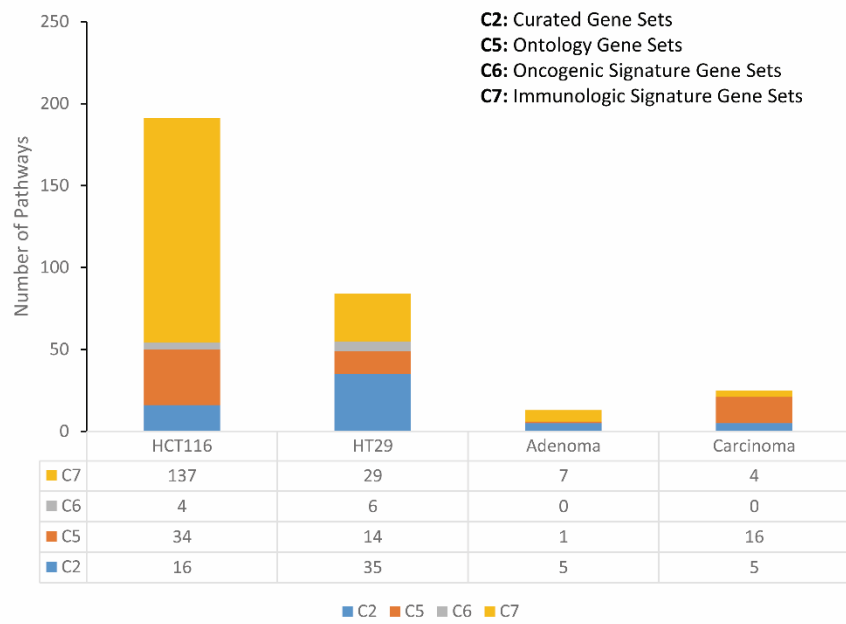

Supplement: Supplementary file 1 [file ijms-25-10367-s001.zip › Figure S3_Upset Plot.pdf]

**F**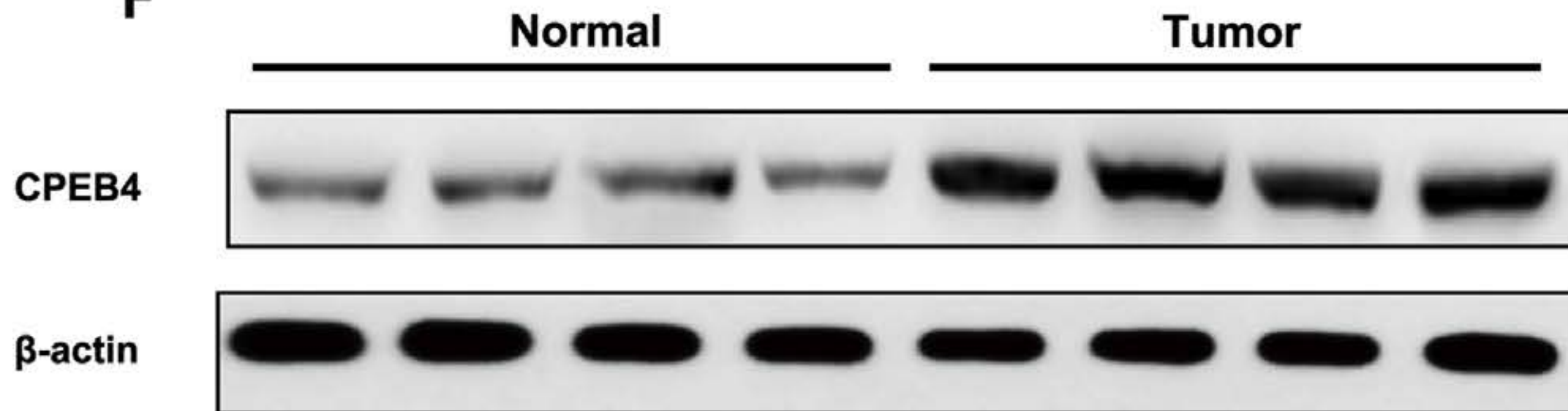**G**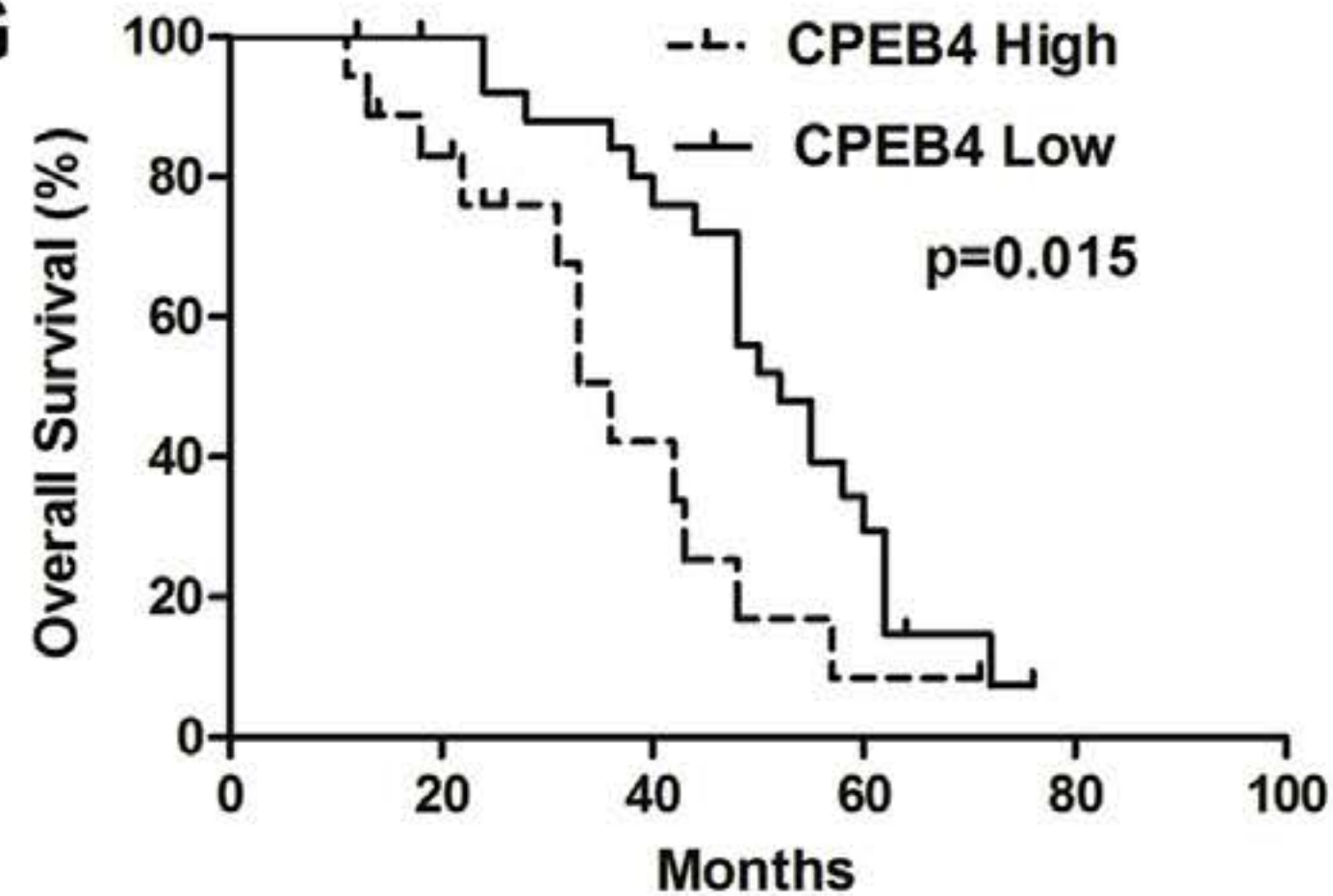

Supplement: Supplementary file 1 [file ijms-25-10367-s001.zip › Figure S4_Western Blot&Survival plot_CPEB4.pdf]
